# Stump appendicitis: challenging diagnosis with serious complication: a case report

**DOI:** 10.1093/jscr/rjad043

**Published:** 2023-04-17

**Authors:** Abdi Alemayehu, Ebisa Woyesa, Desalegn Fekadu

**Affiliations:** Adama Comprehensive Specialized Hospital Medical College, Adama, Ethiopia; Department of Surgery, School of Medicine, Wollega University, Nekemte, Ethiopia; Department of Surgery, School of Medicine, Addis Abeba University, Addis Abeba, Ethiopia

## Abstract

Stump appendicitis is a rare complication of appendectomy because of recurrent inflammation of the residual appendix. The diagnosis is often delayed because of low index of suspicious, which may result in serious complications. Twenty-three-year-old male patient presented with right lower quadrant abdominal pain after 7 month of appendectomy done at a hospital. On physical examination, he has right lower quadrant tenderness and rebound tenderness. Abdominal ultrasound was done with finding of blind-ended tubular noncompressible 2 cm long part of appendix with wall-to-wall diameter of 10 mm. There is also focal defect with surrounding fluid collection. With this finding, perforated stump appendicitis was diagnosed. He was operated with similar intra operative finding. The patient discharged improved after 5 days of hospital. This is first reported case in Ethiopia as far as our search is concerned. Despite past medical history of appendectomy, the diagnosis was made by means of ultrasound scan. Stump appendicitis is a rare but important complication of appendectomy, often misdiagnosed. Prompt recognition is important to avoid serious complications. This pathologic entity should always be kept in mind in case of right lower quadrant pain in patient with previous history of appendectomy.

## INTRODUCTION

Acute appendicitis is the commonest surgical operation, and appendectomy forms a major bulk of the emergency surgery performed in the emergency room. Although stump appendicitis is a complication of appendectomy, it is rare, whereas wound infection, pelvic abscess and adhesive bowel obstruction are more commonly seen among others.

Most clinicians do not consider stump appendicitis as a differential diagnosis when evaluating a patient with right lower abdomen pain who has undergone appendectomy in the past. It is a challenging condition that is usually diagnosed and confirmed by means of imaging, especially by US and CT. A few number of stump appendicitis are reported in the medical literature. Here, a 23-year male with stump appendicitis is described, with a previous history of appendectomy.

## CASE PRESENTATION

A 23-year-old male patient presented with abdominal pain of 4-day duration. The pain increased in intensity 24 hour prior to his arrival to our hospital. The pain gets worse with movement and he has associated some episodes of vomiting. He complains low-grade fever. He has history of open appendectomy 7 month back at the same hospital.

On physical examination, he has rebound tenderness in right lower quadrant in the proximity of a well-healed McBurney’s’s incision scar. Psoas sign was also positive.

His axillary temperature was 38.4°C. Base line investigations showed white blood cells 13.070/mmc with slight neutrophilia at 11.490/mmc. Urine analysis and stool examination were normal.

**Figure 1:**
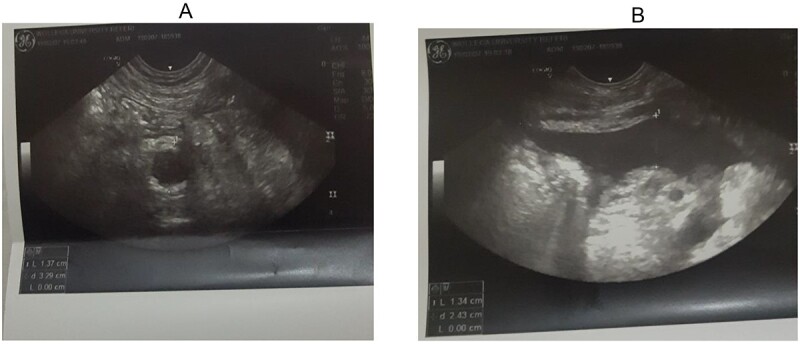
(**A**, **B**) Right lower quadrant ultrasound study showing dilated appendix and right lower quadrant fluid collection.

Abdominal ultrasound was done and there was dilated tubular and blind-ended appendix, which has connection cecum. It is not compressible with outer-to-outer wall diameter of 10 mm as shown in Fig. 1A and B. There was mucosal and wall defect at its distal tip and there was adjacent fluid collection localized to right lower quadrant. With this finding, the diagnosis of stump appendicitis with perforation was made.

On the same day, exploratory laparotomy with a lower midline incision was performed under general anesthesia. The operative findings were inflamed stump of appendix (~2 cm) with perforation at its tip with surrounding abscess collection. Rest of the bowel was normal. Completion appendectomy was performed and the postoperative period was uneventful, and on postoperative Day 5, the patient was discharged (Fig. 1A and B).

## DISCUSSION

Acute appendicitis is the commonest abdominal surgical emergency encountered in clinical practice [1]. It is a clinical diagnosis, and the patient usually presents with periumbilical pain that radiates to the right lower abdomen. But then, it always poses a dilemma when a patient with a history of appendectomy presents with right lower quadrant abdominal pain.

The first appendectomy was performed in 1735 by Claudius Amyand, and in 1886, Reginald Fitz described the clinicopathological features, whereas Rose described stump appendicitis for the first time in 1945 [2]. There is a list of complications following appendectomy, the common ones being wound infection and pelvic abscesses [3].

Stump appendicitis is defined as a rare complication following appendectomy caused by inflammation of the residual portion of the appendix left behind. The clinical presentation of stump appendicitis is similar to that of acute appendicitis [4]. Stump appendicitis has an incidence of 1 in 50 000 cases [5]. The time interval for onset of symptoms can range from 2 weeks to 2 years post-appendectomy. Clinically, these patients have the symptoms and signs similar to appendicitis. Stump appendicitis poses a dilemma if the clinician is not aware of this uncommon presentation.

Generally, during surgery, adequate visualization of the appendix base and the ileocecal region and a stump of <5 mm reduces the risk of stump appendicitis [1]. Although stump appendicitis is thought to be a recent phenomenon that is mainly seen in laparoscopically performed appendectomies, the literature shows that 66% of stump appendicitis occurred after open appendectomy [6].

Stump appendicitis is associated with late diagnosis and therefore has a higher rate of perforation with increased morbidity [7]. The factors leading to stump appendicitis can be either anatomical or surgical. Anatomically, it can be retrocecal in position posing difficulty [8]. While the surgical factors include inadequate identification of the base of appendix because of local inflammation, difficult dissection or leaving a long stump behind because of fear of injury to cecum [9].

A correct preoperative diagnosis of stump appendicitis can be made by ultrasonography and by computed tomography. Ultrasonography can reveal a thickened appendix stump, fluid in the right iliac fossa and edema of caecum. Abdominal sonography has become the method of choice for the diagnosis of acute appendicitis since Puylaert’s technique was adopted [10]. In our case, sonography identified inflammatory changes present in the appendiceal stump and it was capable of revealing perforation with surrounding collection.

While CT scan is said to be superior to USG [11], but in a developing country like Ethiopia, CT scan is not readily available and relatively expensive to be done routinely. CT findings may be similar to those present in acute appendicitis. Contrast enhancing tubular structure arising from the cecum with adjacent fat stranding is usual finding if the appendiceal stump left after appendectomy is long [9]. CT may also demonstrate a pericecal phlegmon or abscess, as well as a thickening of the cecal wall with oral contrast material insinuating into the expected location of appendiceal origin, the so-called ‘arrowhead sign’ [12]. In our case, CT scan is not available and not done.

In developing countries like ours where sophisticated examination like CT, MRI, barium enema or colonoscopy are not routinely available, we think in agreement with other authors, that in most cases the diagnosis of stump appendicitis may be made by ultrasonography alone. Since ultrasound is operated dependent, high index of suspicion and a certain familiarity with sonographic finding are necessary and sufficient prerequisites for an early diagnosis. This can reduce clinical dilemma to diagnose stump appendicitis and more importantly reduce complication related with delay to diagnose. In our case, the patient has stump appendicitis perforation, which is the commonest complication. But ultrasound diagnosis was made preoperatively. So this complication may be explained by late patient presentation, 4 days after onset of pain [13].

The treatment of choice is completion appendectomy, either open or laparoscopically [14]. Sometimes ileocolic resection may be required depending on the clinical presentation and inflammation around ileocecal region [15].

## CONCLUSION

In conclusion, stump appendicitis is a rare but serious complication of appendectomy, often confused with other conditions. The prevalence and the incidence of stump appendicitis have been increasing in recent years, probably because of the increased use of laparoscopic approach to appendectomy. Prompt recognition is important to lead to early treatment, thus avoiding serious complications. It must be clear that the history of prior appendectomy does not rule out the possibility of a stump appendicitis. High degree of suspicion can help to make a correct diagnosis and a safe treatment. Therefore, radiologist should be familiarized and aware of this rare but important finding for timely diagnosis. Clinicians should also always keep in mind the possibility of this complication as the cause of right lower quadrant pain.

## CONFLICT OF INTEREST STATEMENT

None declared.

## FUNDING

None.
